# Fournier’s Gangrene in a Young, Morbidly Obese Patient Without Diabetes

**DOI:** 10.7759/cureus.104387

**Published:** 2026-02-27

**Authors:** Thomas S Varghese, Julie M Riley, Jack Campbell

**Affiliations:** 1 Department of Surgery, Division of Urology, University of Missouri School of Medicine, Columbia, USA

**Keywords:** actinomyces funkei, fournier's gangrene, morbid obesity, necrotizing fasciitis, urologic emergency

## Abstract

Fournier’s gangrene (FG) is a rapidly progressive form of necrotizing fasciitis involving the external genitalia, perineum, and perianal region. We present a case of FG in a morbidly obese patient (BMI > 40) without a known history of diabetes, malignancy, or immunodeficiency. The patient was treated with aggressive surgical debridement, broad-spectrum antibiotics, and early closure, achieving full recovery. This case highlights obesity as an independent risk factor for FG, which has significant ramifications for the future, given obesity rates in young Americans.

## Introduction

Fournier’s gangrene (FG) is a dangerous condition, with a mortality rate of 20-30%, notably worsening up to 90% with delays in treatment [1,2]. Despite its significant risk for morbidity and mortality with any delay in treatment, FG often avoids clinical detection early in a patient’s course. The patient presented herein had a past medical history significant for morbid obesity (BMI > 40), but no history of diabetes mellitus, urethral stricture disease, malignancy, or immunodeficiency. While obesity is an oft-noted risk factor for FG, other issues are usually present in patients who develop FG [1,2]. Diabetes mellitus is often recognized as the primary risk factor in the genesis of FG, but is not always present, as this case will illustrate [1,2]. While FG is frequently described as polymicrobial with a broad range of both anaerobic and aerobic bacteria, *Actinomyces funkei* was a rare and unusual bacterium isolated in this case [3,4].

## Case presentation

The patient is a 32-year-old male with a past medical history of morbid obesity (BMI of 42.3) who presented to the emergency department with the chief complaint of scrotal swelling and pain. Approximately one week before arrival, the patient popped what he believed to be a boil or abscess in his right groin. He reported that four days later, he began to have a fever (101 °F), dyspnea, and progressively worsening fatigue in conjunction with scrotal swelling, pain, and erythema. He noted significant nausea and vomiting in the three days prior to arrival, as well as constipation and increased work of breathing with a dry cough. He denied any urinary concerns and rated his pain as 10 out of 10.

On physical exam, the patient had severe right-sided groin swelling with overlying erythema and significant tenderness to palpation. The patient was tachycardic (110 beats per minute) and hypertensive (143 mmHg/74 mmHg) but stable overall.

**Figure 1:**
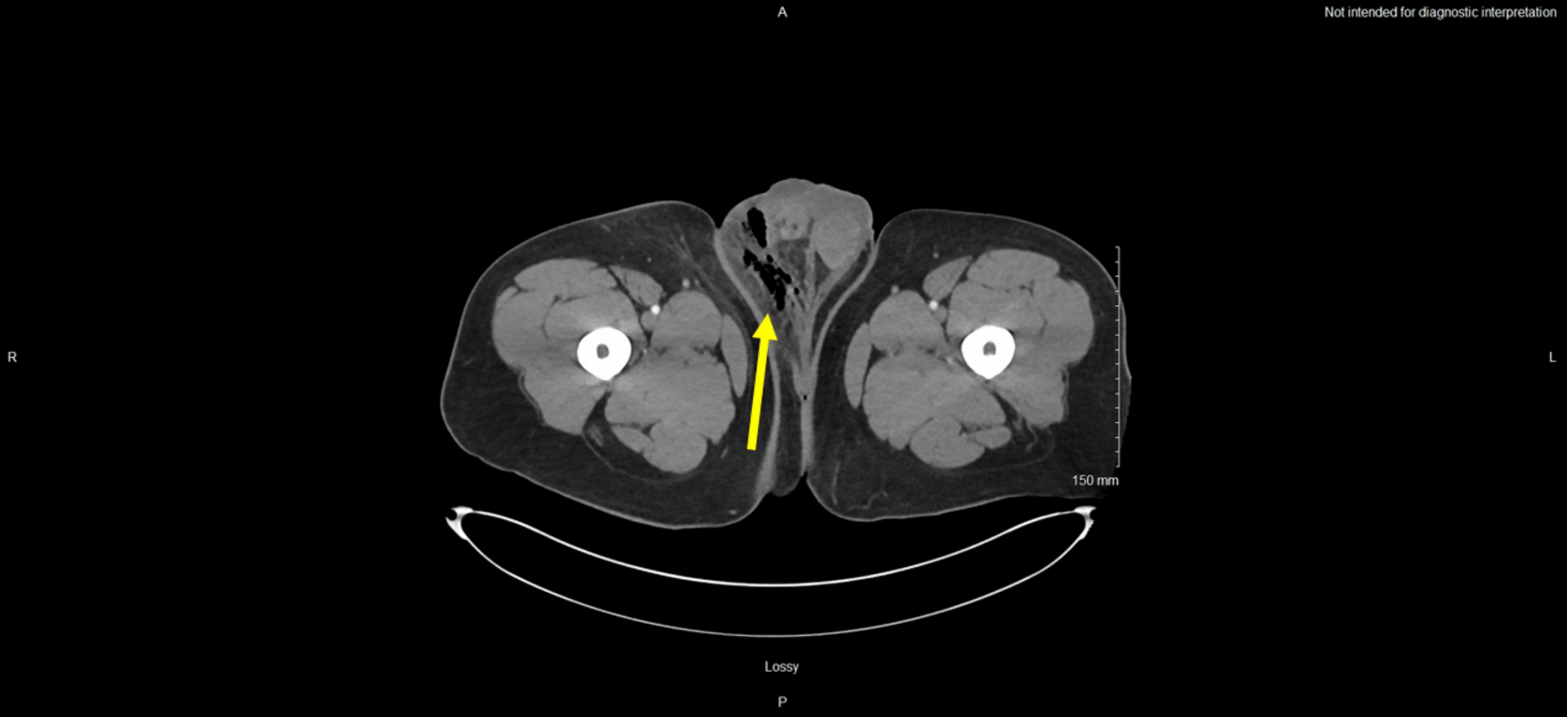
Axial view of the contrast-enhanced CT scan demonstrating soft tissue edema and subcutaneous emphysema involving the right hemiscrotum

**Figure 2:**
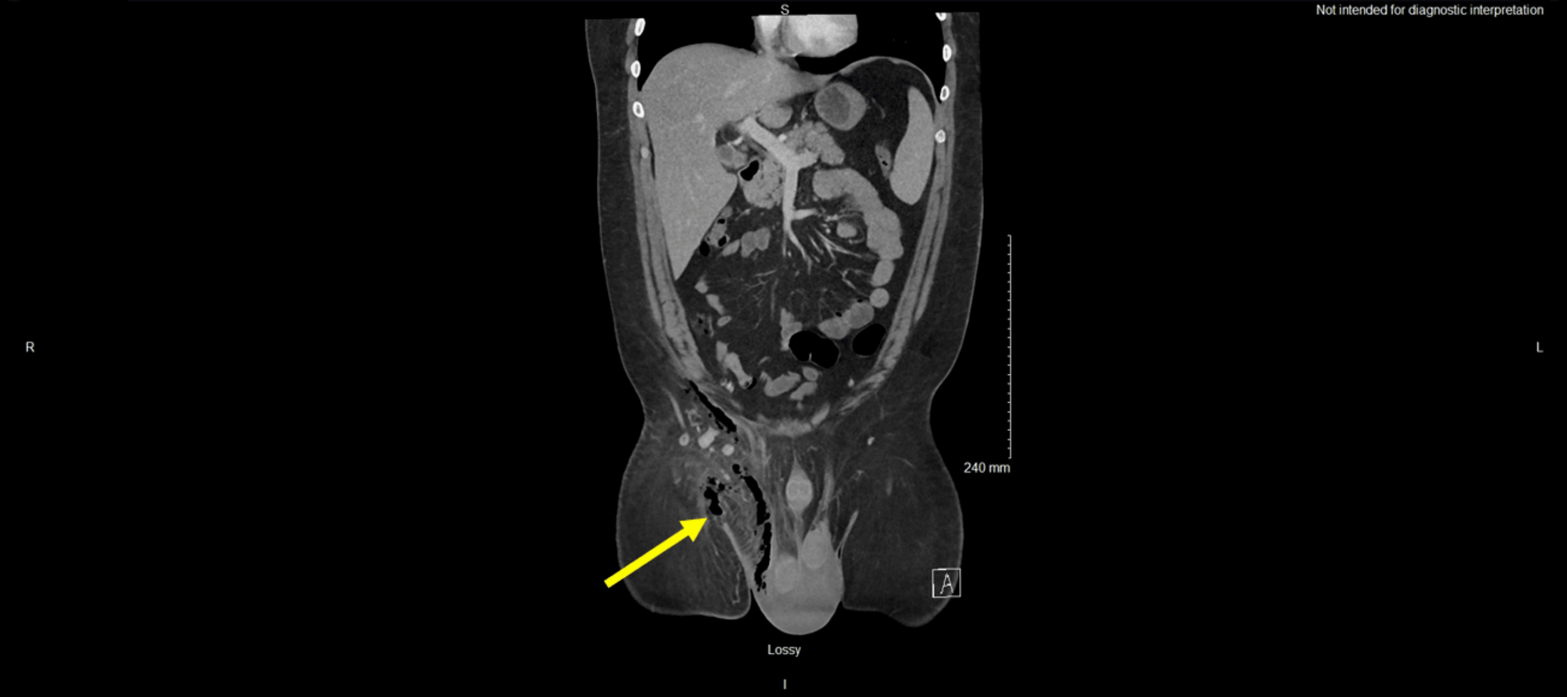
Coronal view of contrast-enhanced CT image demonstrating extension of subcutaneous gas from the right hemiscrotum into the inguinal region

After collecting vital signs, laboratory tests, including CBC, comprehensive metabolic panel (CMP), lactic acid, liver function tests, and blood cultures were ordered. White blood cell count and lactic acid were elevated at 23.07 × 10 (9)/L and 4.7 mmol/L, respectively. The patient had a mild acute kidney injury with a creatinine of 1.5 mg/dL, and hemoglobin A1c was notably normal at 5.2%. Lab findings and corresponding reference ranges are listed in Table 1. Imaging studies included a point-of-care scrotal ultrasound and a CT abdomen and pelvis. Scrotal ultrasound was performed shortly after arrival, which revealed a hyperemic and edematous scrotum with subcutaneous air, concerning for infection/Fournier’s gangrene. Contrast-enhanced CT scan was also suggestive of FG with extensive soft tissue edema, inflammatory changes, and subcutaneous gas extending from the right hemi-scrotum into the groin (Figures 1, 2).

**Table 1:** Selected laboratory values on presentation, with corresponding reference ranges

Lab Test	Result	Reference Range
White Blood Cell Count	23.07 x10(9)/L	3.5-10.5 x10(9)/L
Neutrophils	19.56 x10(9)/L	1.7-7 x10(9)/L
Lactic Acid	4.7 mmol/L	0.5-2.2 mmol/L
Creatinine, Standardized	1.5 mg/dL	0.7-1.2 mg/dL
Hemoglobin A1c	5.20%	4.0-5.6%

The patient was taken to the operating room for emergent incision, drainage, and debridement of all necrotic tissue, which included most of the perineum, right hemi-scrotum, and right thigh to right inguinal skin. He was treated with broad-spectrum antibiotics, including vancomycin, clindamycin, and piperacillin-tazobactam. There was no rectal, urethral, or testicular involvement. The patient did well postoperatively and did not require intensive care. After two additional takebacks for minor debridement, the patient was closed with local tissue rearrangement primarily by the reconstructive urology team.

Cultures obtained from the wound demonstrated multiple anaerobic species, including the predominant *Actinomyces funkei*. Following initial cultures, vancomycin and clindamycin were discontinued, and linezolid was started in conjunction with piperacillin-tazobactam based on culture sensitivities. On discharge, the patient was started on oral linezolid and amoxicillin-clavulanic acid for two weeks following closure of the wound. Infectious disease recommended six months of treatment with long-term antibiotic therapy for *Actinomyces* infection based on sensitivities.

## Discussion

This case is an unusual presentation of Fournier’s gangrene in an obese individual in the absence of diabetes, immunodeficiency, advanced age, chronic alcoholism, or malignancy, several of the major risk factors identified for FG [1]. The case demonstrates obesity as a predisposing risk factor and *Actinomyces funkei *as a rare causative bacterium in FG.

FG is most frequently characterized as a community-acquired polymicrobial infection whose causative pathogens commonly include the following bacterial species: *Staphylococcus, Streptococcus, Bacteroides, Candida, Prevotella, Escherichia coli,* and others [3,4]. In recent years, some studies have noted an increased frequency of Methicillin-resistant *Staphylococcus aureus* (MRSA) and multidrug-resistant organism (MDRO) infections leading to FG, highlighting the necessity for the rapid administration of broad-spectrum antibiotics with anti-MDRO activity [5].

While described, a culture positive for *Actinomyces funkei*, among other unspecified bacteria, was a rare, unusual aspect of this case and partially responsible for the long course of antibiotics required for recovery. Human actinomycosis is a chronic, granulomatous disease caused by the genus Actinomyces, of which approximately 25 species have been described from human sources, including *Actinomyces funkei* [6]. Actinomyces species are notoriously implicated in abscess formation throughout the body, including the mouth, lungs, gastrointestinal tract, and skin structures. Prolonged treatment is often required due to actinomycosis’s chronic and invasive nature, characteristics acquired through fimbria and biofilm capabilities [7]. 

*Actinomyces funkei* infections are often polymicrobial and difficult to isolate, which often leads to a delayed diagnosis [7]. A literature review revealed one case report describing FG caused by *Actinomyces funkei,* *Fusobacterium gonidiaformans*, and *Clostridium hathewayi*. Tena et al. noted the difficulty in identifying *Actinomyces funkei*, especially in polymicrobial infections such as FG [8]. Strains of Actinomyces frequently struggle to grow in culture and are often missed in attempts at detection. Conventional identification tests and biochemical kits are generally insufficient for reliably detecting the presence of Actinomyces, leading to false-negative results [9].

*Actinomyces funkei *is an aero-tolerant species and is believed to be a member of the normal skin and mucous membrane flora, as are other Actinomyces species [9]. While actinomycosis remains relatively rare, recent advancements in microbiological techniques allow for the detection and increased awareness of the genus [9].

Current literature indicates that obesity is present in a substantial proportion of patients with Fournier's gangrene, with one manuscript indicating that increased BMI was associated with an increased risk of 30-day mortality; age, white blood cell count, and decreased platelets were similarly associated [10]. In our literature review, diabetes mellitus was found to be the most prevalent predisposing risk factor for FG, ranging from a prevalence of 36.4% to 76.9% among reviewed cases [5,11,12]. Besides diabetes, other risk factors frequently identified with increased mortality risk included socioeconomic status, greater than one debridement, and malnutrition [1]. The underlying principle of these risk factors is the establishment of a favorable infectious environment. Obesity has the potential for poor perineal hygiene, which could be one explanation for its association with FG [1]. However, it should be noted that the patient appeared quite meticulous in maintaining personal hygiene, which further contributes to the case’s unusual presentation.

The patient met several qualifications that could indicate a worse outcome, including the criteria for morbid obesity with a BMI of 42.3 and multiple instances of surgical debridement. However, the patient described was younger, at age 32, than most patients represented in the current FG literature, as the average age of presenting patients is 50.9 years according to Chernyadev et al. This case serves as a warning of a potential spike in FG cases with growing obesity rates, especially in younger populations. The Centers for Disease Control (CDC) observed a 9.4% prevalence of severe obesity (BMI >40) from 2021 to 2023 [13]. This stands in stark contrast to the 1970s, when the rate of severe obesity was 1% or less for all ages combined [14]. This radical increase in severe obesity in younger populations may translate to a drastic increase in FG cases.

In a patient without the classic primary risk factors of FG, a healthy clinical suspicion for FG in patients with compatible symptoms is essential to prevent the significant morbidity and mortality that come with delayed treatment in this condition. Early diagnosis, aggressive surgical intervention, and broad-spectrum antibiotics with a subsequent tailored approach form the foundation of treating FG patients in the acute and long-term setting. Early involvement of a multidisciplinary team was essential to support this critically ill patient. With his improvement, the patient is expected to have a full recovery, albeit with moderate cosmetic changes.

## Conclusions

Fournier's gangrene (FG) is a fulminant, necrotizing fasciitis of the external genitalia and perianal and perineal regions with lethal potential, particularly with delays in treatment. When identified in FG, *Actinomyces funke*i may be difficult to detect and requires long-term, broad-spectrum antibiotic treatment. Amid rising rates, obesity should be recognized as a significant risk factor in the development of FG, even in the absence of classical risk factors like diabetes, malignancy, chronic alcoholism, and immunodeficiency.

Healthy clinical suspicion for FG in nontraditional FG patients is essential to the early diagnosis and treatment of this aggressive infection. Treatment includes urgent surgical debridement and immediate broad-spectrum antibiotic therapy. Early involvement of a multidisciplinary team, including urology, acute care surgery, emergency medicine, and infectious disease, among others, is essential to effective care for patients with FG.
